# Rapid and efficient transfer of the T cell aging marker CD57 from glioblastoma stem cells to CAR T cells

**DOI:** 10.18632/oncoscience.164

**Published:** 2015-05-15

**Authors:** Xuekai Zhu, Gabriele Niedermann

**Affiliations:** ^1^ Department of Radiation Oncology, University Hospital Freiburg, Freiburg, Germany; ^2^ German Cancer Consortium (DKTK), Freiburg, and German Cancer Research Center (DKFZ), Heidelberg, Germany

**Keywords:** CD57, trogocytosis, chimeric antigen receptor, cancer stem cells, glioblastoma

## Abstract

Adoptive transfer of T cells expressing chimeric antigen receptors (CARs) holds great promise for cancer treatment. We recently developed CAR T cells targeting the prototypic cancer stem cell marker AC133 and showed that these CAR T cells killed AC133+ glioblastoma stem cells (GBM-SCs) ***in vitro*** and inhibited the growth of brain tumors initiated from GBM-SCs in xenograft mouse models ***in vivo***. Upon coincubation with GBM-SCs, we observed strong upregulation of the T cell aging marker CD57, but other phenotypical or functional changes usually associated with terminal T cell differentiation could not immediately be detected. Here, we provide evidence suggesting that CD57 is rapidly and efficiently transferred from CD57+ GBM-SCs to preactivated T cells and that the transfer is greatly enhanced by specific CAR/ligand interaction. After separation from CD57+ tumor cells, CD57 epitope expression on T cells decreased only slowly over several days. We conclude that CD57 transfer from tumor cells to T cells may occur in patients with CD57+ tumors and that it may have to be considered in the interpretation of phenotyping results for tumor-infiltrating lymphocytes and perhaps also in the characterization of tumor-specific T cells from tumor or lymph node homogenates or peripheral blood mononuclear cells.

## INTRODUCTION

Tumor-specific chimeric antigen receptors (CARs) are recombinant molecules consisting of an antibody moiety specific for a tumor cell surface antigen fused to intracellular signaling domains of the physiological T cell receptor (TCR) and of costimulatory receptors [1]. Engineered T cells expressing CARs are therefore capable of recognizing tumor cells in a major histocompatibility complex (MHC)-independent manner, resulting in T cell activation and killing of bound target cells. CAR T cells have shown extraordinarily high activity against CD19+ hematological malignancies [2,3], and great efforts are undertaken to develop CAR T cells for the treatment of other hematological malignancies and solid tumors.

We recently reported on CAR T cells recognizing the cancer stem cell (CSC) antigen AC133, an N-glycosylation-dependent, stem cell-specific epitope of CD133, marking CSCs of many tumor entities including the highly malignant glioblastoma multiforme (GBM) [4,5]. These CAR T cells killed AC133+ tumor cells, including patient-derived glioblastoma stem cells (GBM-SCs), *in vitro* and in orthotopic tumor models *in vivo* [6].

The functionality of conventional T cells is impaired by the activation of negative regulatory immune checkpoints, T cell exhaustion and terminal T cell differentiation, i.e., processes that are induced by repetitive chronic TCR-mediated stimulation [7-9]. The functionality of CAR T cells may also be compromised by these factors [10,11]. We therefore analyzed the expression of T cell surface markers indicative of the respective functional impairments.

Upon coincubation of CAR T cells with patient-derived GBM-SCs, we consistently observed an upregulation of CD57 [6], a terminally sulfated carbohydrate epitope best known as a marker for terminally differentiated, end-stage T cells [12,13]. Truly terminally differentiated T cells lose their proliferative capacity and expression of the positive costimulatory molecules CD27 and CD28, which usually correlates with loss of telomerase activity and critical shortening of the telomeres; in addition, they upregulate the cytotoxic granule molecules granzyme B and perforin [7,12]. However, we neither observed a loss of the proliferative capacity of CD57+ CAR T cells upon subsequent short-term re-exposure to AC133+ target cells nor did we observe the downregulation of CD27 or CD28 [6]. Wu *et al*. [14] recently described a subpopulation of melanoma-specific CD8+ tumor-infiltrating lymphocytes (TILs) with hybrid phenotypic and functional properties of both an early effector-memory cell and a terminally differentiated effector cell co-expressing CD27, CD28, and CD57. These CD57+ T cells were classified as incompletely differentiated T cells. This TIL subpopulation lost CD27 expression and gained expression of the cytotoxic granule protein perforin after subsequent anti-CD3/anti-CD28-mediated stimulation, indicating transition to the terminally differentiated state.

We previously observed that CD57 expression on CAR T cells increased very rapidly (in less than 2 h) and only upon coculture with patient-derived GBM-SCs, but not with conventional U251 glioma cells [6]. Since CD57 has been found on neuroblastoma and Ewing sarcoma cells with aggressive CSC-like features [15,16], we investigated if CD57 was expressed by the GBM-SCs. CD57 was indeed expressed on all patient-derived GBM-SC lines studied, but it was not lost upon their differentiation, which let us conclude that CD57 is not a *bona fide* CSC marker for GBM [6].

The observation that CD57 increased on CAR T cells in less than a few hours and only upon encounter with CD57+ target cells suggested that proteins expressing CD57 carbohydrate epitopes may simply be transferred from CD57+ tumor cells to CAR T cells. In our previous work, we tried to prove the transfer of CD57+ proteins to T cells after prelabeling of CD57 on the tumor cells with a fluorescently labeled anti-CD57 antibody [6]. However, antibody binding may have hindered the intercellular transfer of CD57+ proteins onto the T cells. We have now obtained more evidence suggesting that CD57 is indeed rapidly and efficiently transferred from CD57+ tumor cells to prestimulated T cells and that this process is greatly enhanced by the specific CAR/ligand interaction.

**Figure 1:**
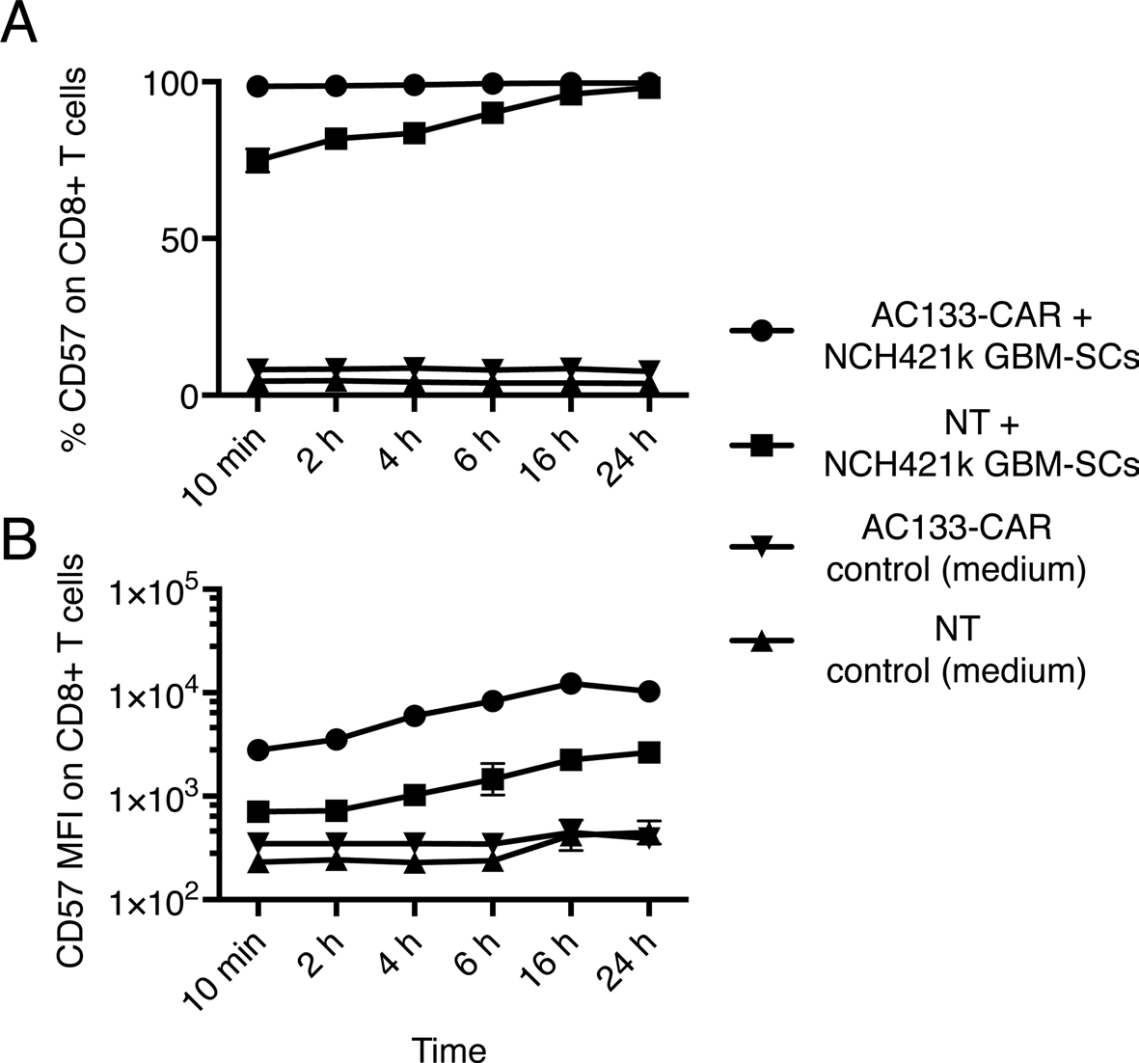
Kinetics of the gain of CD57 expression on T cells upon coincubation with CD57+ AC133+ NCH421k GBM-SCs AC133-CAR T cells or NT anti-CD3-prestimulated T cells were cocultured with NCH421k GBM-SCs at a ratio of 1:1 for the indicated periods of time. Thereafter, the percentage of CD57+ cells among the CD8+ T cells (**A**) and the CD57 expression level on the T cells (**B**) were assessed by flow cytometry (for details of Materials and Methods, see Ref. [6]). On the vast majority of AC133-CAR or NT T cells, CD57 expression was already detected within 10 min of coincubation; however, the per cell expression level was considerably higher on AC133-CAR than on NT T cells. Data are representative of one out of three experiments, measured in triplicate, and are presented as mean ± SD. MFI, mean fluorescence intensity.

We first evaluated the detailed kinetics of CD57 upregulation on AC133-specific CAR T cells upon coculture with CD57+ tumor cells. As shown in Figure 1, strong upregulation of CD57 on the T cells occurred very rapidly, within 10 min, regardless of whether AC133-CAR or nontransfected (NT) prestimulated control CD8+ T cells were cocultured with AC133+ CD57+ NCH421k GBM-SCs. This ruled out the possibility that CD57 expression resulted from transcriptional and translational changes in the T cells, at least at the beginning of the coincubation period. Rather, it was likely the result of a direct transfer of the CD57+ proteins from the tumor cells to the T cells. The specific CAR/ligand interaction strongly enhanced the transfer. Not only was the percentage of CD57+ T cells higher when CAR T cells were incubated with NCH421k GBM-SCs compared to NT T cells (Figure 1A), but also the mean fluorescence intensity (MFI) was higher (Figure 1B). The MFI for CD57 expression increased 7–26-fold for the AC133-CAR T cells while it increased only 2–6-fold for the NT control T cells at different time points of coincubation with AC133+ CD57+ tumor cells.

**Figure 2:**
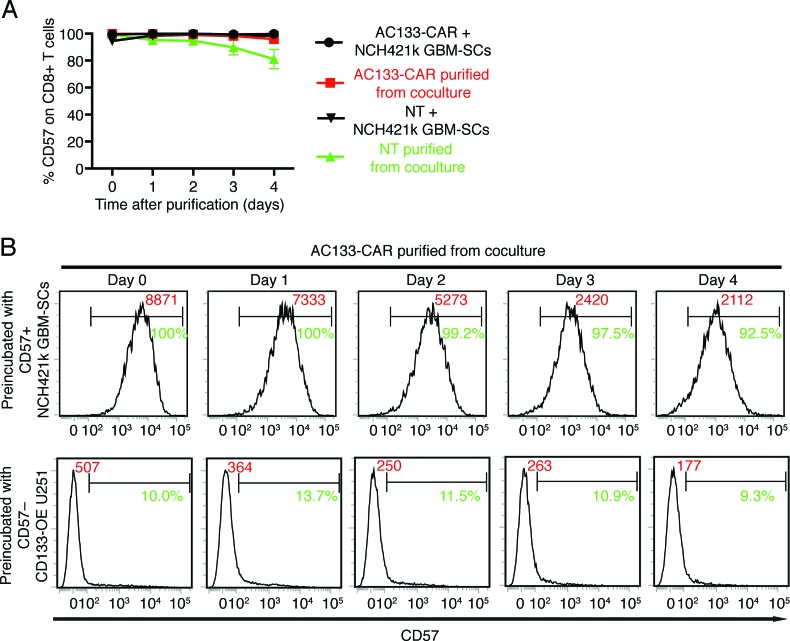
CD57 epitope expression on T cells decreases only slowly over a period of several days after separation from CD57+ tumor cells AC133-CAR T cells or NT control T cells were cocultured with CD57+ AC133+ NCH421k GBM-SCs or CD57– AC133+ CD133-OE U251 cells at a ratio of 1:1 for 4 h. Thereafter, CD8+ T cells were purified from the coculture using the EasySepTM Human CD8 Positive Selection Kit (STEMCELL Technologies). After separation, the T cells were cultured in T cell medium for the indicated periods of time and CD57 expression was then determined by flow cytometry (for details of Materials and Methods, see Ref. [6]). (**A**) Percentage of CD57+ cells among the CD8+ T cells at the indicated time periods after separation from the CD57+ tumor cells. Data are representative of one out of three experiments, measured in triplicate, and are presented as mean ± SD. (**B**) Representative flow cytometry results. The percentage of CD57+ cells among the CD8+ CAR T cells is indicated in green; the MFI is given in red.

Since transfer of CD57 from tumor cells to T cells may affect the phenotyping of TILs isolated from tumor or lymph node homogenates or of tumor-specific T cells from peripheral blood mononuclear cells (PBMCs), we wanted to find out for how long CD57 can be detected on T cells after separation from the CD57+ tumor cells. As shown in Figure 2, 4 days after the separation of the AC133-specific CAR or NT control T cells from the AC133+ CD57+ NCH421k GBM-SCs, more than 90% of the CAR T cells or 80% of the NT cells were still CD57+ (Figure 2A). Although the MFI for CD57 expression on the CAR T cells had slowly and continuously dropped after separation from the tumor cells, CD57 was still significantly expressed after 4 days (Figure 2B, top panels). Of note, during this 4-day period, no strong CD57 expression was detected on the T cells when they had been preincubated with CD57– AC133+ tumor cells (Figure 2B, bottom panels); it is therefore unlikely that the high CD57 expression level on the T cells during the 4-day period following incubation with the CD57+ tumor cells was due to endogenous expression by the T cells. Taken together, these results suggest that CD57 transfer from CD57+ tumor cells to T cells could indeed contribute to CD57 expression on tumor-specific T cells isolated from tumor or lymph node homogenates or PBMCs of patients with CD57+ tumors.

**Figure 3:**
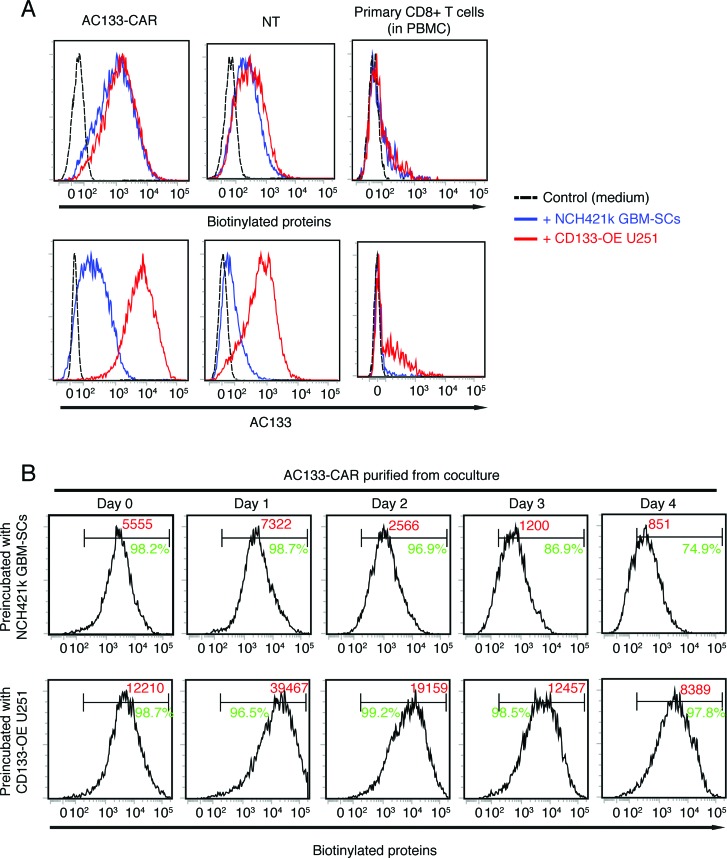
Transfer of biotinylated proteins and AC133 from glioma cells to preactivated CD8+ T cells For biotinylation of AC133+ glioma cells, either NCH421k GBM-SCs or CD133-OE U251 cells were incubated with EZ-Link Sulfo-NHS-LC-Biotin (Pierce™) following the manufacturer's protocol. AC133-specific CAR T cells, NT anti-CD3-prestimulated T cells, or PBMCs were cocultured with biotinylated tumor cells at a ratio of 1:1 for 4 h. Thereafter, expression levels of biotinylated proteins (after staining with PE-streptavidin, Biolegend) or AC133 (after staining with anti-AC133 as described in Ref. [6]) were determined by flow cytometry (**A**). (**B**) To determine for how long the transferred biotinylated proteins can be detected on the separated T cells, CD8+ T cells were isolated from the coculture and further cultured in T cell medium for 4 days, as described in the legend to Figure 2. At the indicated periods of time after separation, the percentage of biotin+ cells among the CD8+ CAR T cells was determined by flow cytometry and is indicated in green; the MFI is given in red. Data presented are representative of at least three independent experiments.

The aforementioned findings suggest that T cells might gain CD57 via trogocytosis [17,18], a process where membrane fragments are directly transferred between cells within a few minutes and are detectable on the recipient cells for a considerable period of time [19-21]. Via trogocytosis, a wide range of proteins are usually transferred between cells. Therefore, we wanted to figure out if membrane proteins other than CD57 were transferred between tumor cells and T cells. For this purpose, we studied the transfer of biotinylated membrane proteins or AC133. In these experiments, AC133-specific CAR T cells, NT control T cells, or primary CD8+ T cells from PBMCs were incubated with biotinylated NCH421k GBM-SCs or CD133-overexpressing U251 glioma cells (CD133-OE U251), which exhibit 10–15-fold higher AC133 expression than NCH421k GBM-SCs [6,22]. As shown in Figure 3A, we indeed observed that not only AC133 but also biotinylated proteins were transferred from glioma cells to CD8+ T cells. The transfer of biotinylated membrane proteins (Figure 3A, top panels) was influenced by two factors: (i) the activation status of the T cells (high transfer only occurred onto anti-CD3-prestimulated CAR T and NT T cells but not onto primary CD8+ T cells) and (ii) the expression of the tumor-specific CAR (much more biotinylated proteins were transferred onto AC133-CAR T cells than onto NT T cells). The transfer of AC133 (Figure 3A, bottom panels) was also strongly influenced by the AC133 expression level on the tumor cells (much more AC133 was transferred from CD133-OE U251 cells than from NCH421k GBM-SCs). An influence of the molecule level on the tumor cells was also observed in our previous report [6] for CD57 upregulation on T cells (higher CD57 expression on GBM-SCs conferred higher upregulation of CD57 on CAR T cells after coincubation). Similar to what we observed for CD57 (see Figure 2), the biotinylated proteins transferred from the tumor cells to the CAR T cells were detectable for at least 4 days on the T cell surface after separation from the tumor cells (Figure 3B).

As shown in Figure 3A, only a minor fraction of the primary CD8+ T cells acquired biotinylated proteins and AC133 from the tumor cells. To figure out if membrane proteins were also transferred onto naïve CD8+ T cells, we compared the amount of biotinylated proteins and AC133 between naïve T cells (CD45RA+ CD62L+) and memory T cells (CD45RO+) upon coincubation of PBMCs with the tumor cells. Both naïve and memory T cells acquired proteins from tumor cells, although the memory T cells acquired more proteins (data not shown).

**Figure 4:**
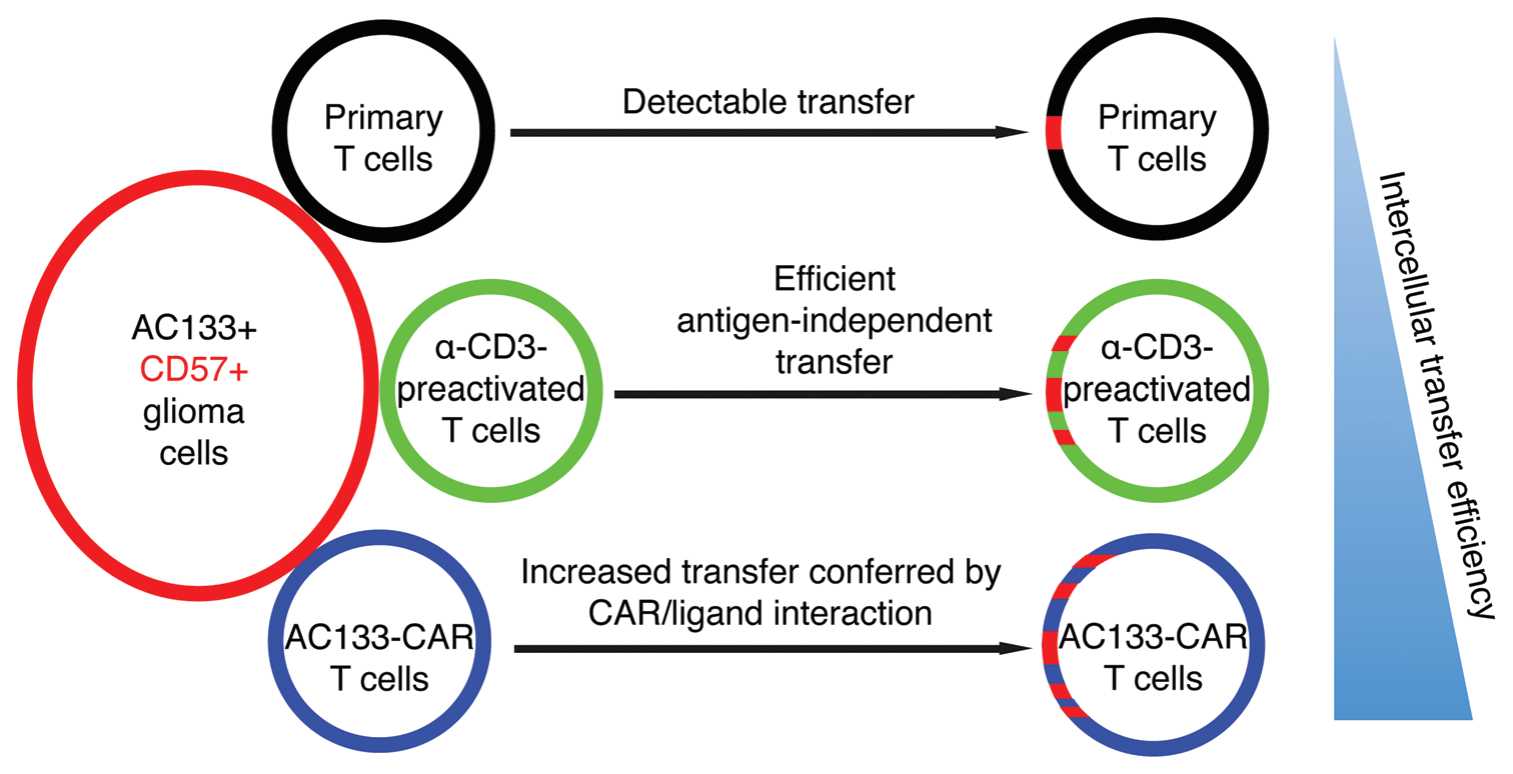
Schematic representation of the intercellular transfer efficiency of membrane proteins from glioma cells to CD8+ T cells The experimental data suggest that primary CD8+ T cells acquire only low amounts of protein from tumor cells. Transfer to polyclonal T cells prestimulated with anti-CD3 (as described in Ref. [6]) is much higher and is further considerably increased by specific CAR/ligand interaction.

The data presented here suggest an extremely rapid and efficient transfer of CD57 from AC133+ CD57+ tumor cells to AC133-specific CAR T cells. Together with our previous findings [6], the data suggest that CD57 expression on tumor-specific T cells does not always indicate terminal or near-terminal T cell differentiation and that it could, at least in part, also simply reflect a recent encounter with CD57+ tumor cells. Furthermore, the data strongly suggest that the CD57 transfer is greatly increased by specific, receptor-mediated interaction between the tumor cells and T cells (Figure 4). This transfer likely occurs through trogocytosis. For conventional T cells, evidence of membrane protein transfer from antigen-presenting cells or tumor cells by trogocytosis facilitated by specific peptide-MHC/TCR interaction has already been reported [23]. We now report evidence that the intercellular transfer of membrane proteins to T cells is also greatly facilitated by specific CAR/CAR target interaction. However, CD57 was also transferred to anti-CD3-prestimulated control T cells, albeit to a lesser extent (Figure 4). Transfer to primary or naïve T cells was negligible. Intercellular transfer of CD57 from tumor cells to T cells may have to be considered in the interpretation of phenotyping results for TILs from CD57+ tumor entities such as brain tumors [6,24-26] and neuroectodermal tumors (including neuroblastoma, Ewing's sarcoma, and melanoma [15,16,27,28]), at least in case of *in situ* analyses such as immunohistochemical analyses. Since the CD57 levels on T cells only slowly decreased over several days after separation from CD57+ tumor cells, the intercellular transfer may even contribute to CD57 expression on effector/memory T cells in PBMCs from patients with CD57+ tumors.
